# NLRP3 Inflammasome: Checkpoint Connecting Innate and Adaptive Immunity in Autoimmune Diseases

**DOI:** 10.3389/fimmu.2021.732933

**Published:** 2021-10-11

**Authors:** Yiwen Zhang, Wenlin Yang, Wangen Li, Yunjuan Zhao

**Affiliations:** ^1^ Department of Dermatology, The Second Affiliated Hospital of Guangzhou Medical University, Guangzhou, China; ^2^ Department of Endocrinology, The Second Affiliated Hospital of Guangzhou Medical University, Guangzhou, China

**Keywords:** NLRP3, autoimmune diseases, inflammatory bowel disease, rheumatoid arthritis, type 1 diabetes, systemic lupus erythematosus

## Abstract

Autoimmune diseases are a broad spectrum of human diseases that are characterized by the breakdown of immune tolerance and the production of autoantibodies. Recently, dysfunction of innate and adaptive immunity is considered to be a key step in the initiation and maintenance of autoimmune diseases. NOD-like receptor family pyrin domain containing 3 (NLRP3) inflammasome is a multimeric protein complex, which can detect exogenous pathogen irritants and endogenous danger signals. The main function of NLRP3 inflammasome is to promote secretion of interleukin (IL)-1β and IL-18, and pyroptosis mediated by caspase-1. Served as a checkpoint in innate and adaptive immunity, aberrant activation and regulation of NLRP3 inflammasome plays an important role in the pathogenesis of autoimmune diseases. This paper reviewed the roles of NLRP3 inflammasome in autoimmune diseases, which shows NLRP3 inflammasome may be a potential target for autoimmune diseases deserved further study.

## Introduction

**Figure 1:**
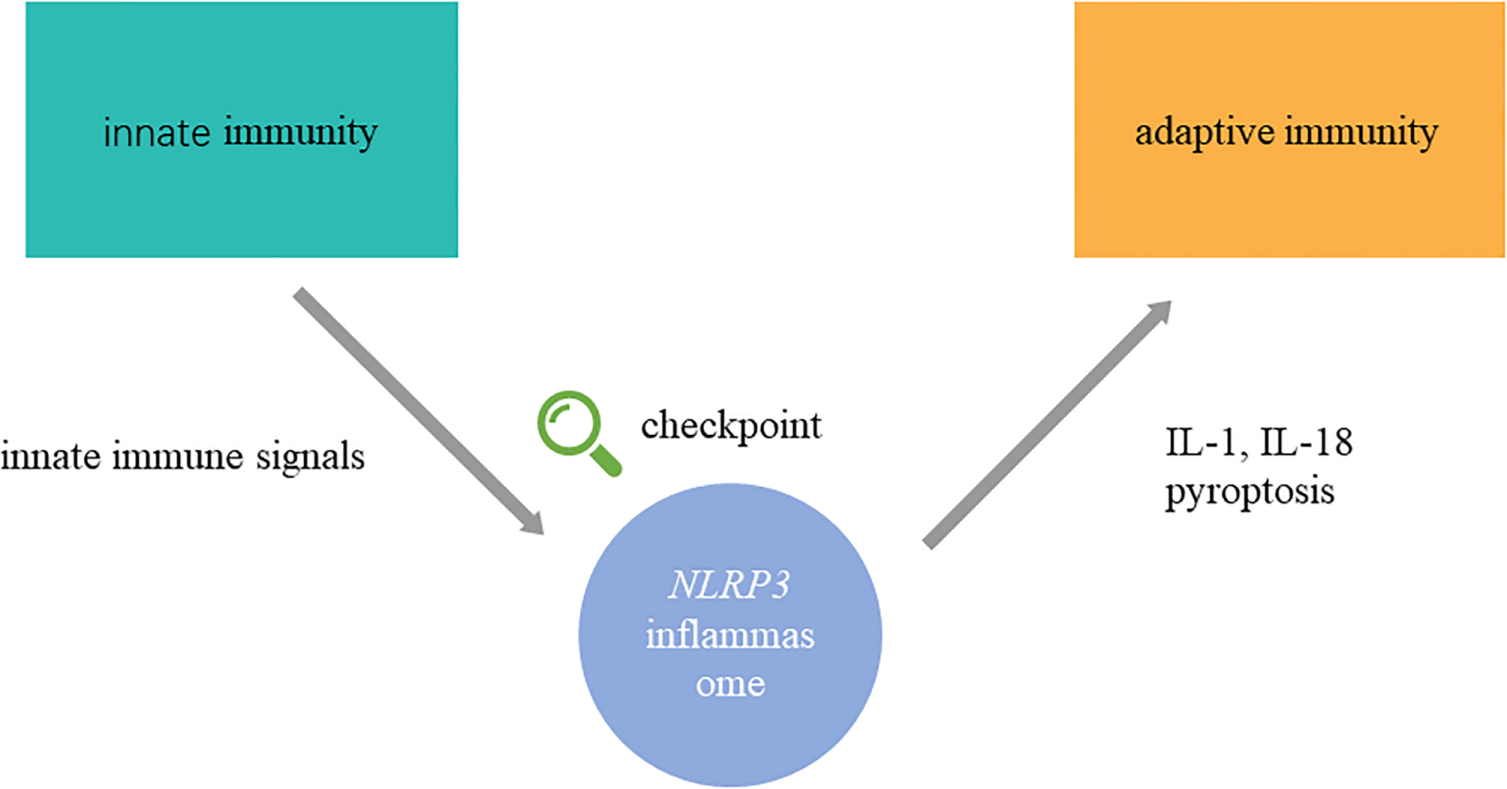
NLRP3 inflammasome connects innate and adaptive immunity in autoimmune diseases. NLRP3 inflammasome is activated abnormally in autoimmune diseases. In the upstream process, exogenous pathogen irritants and endogenous danger signals initiate assembly of NLRP3 inflammasome. In the upstream process, IL-1β, IL-18, and pyroptosis, which are modulated by NLRP3 inflammasome, regulate adaptive immune response. Namely, NLRP3 inflammasome might participate in the transition from innate immunity to adaptive immunity in the pathogenesis of autoimmune diseases as a checkpoint. The detailed process of activation, assembly, regulation, and effects on adaptive immunity are shown in **Figure 2**.

Autoimmune diseases are characterized by self-reactive cells and the overproduction of autoantibodies, which are led by the breakdown of immunological tolerance and aberrant autoreactive immune responses (1). Autoimmune diseases include organ-specific autoimmune diseases, such as type 1 diabetes, autoimmune thyroid diseases, and rheumatoid arthritis, and systemic autoimmune diseases, such as systemic lupus erythematosus and systemic sclerosis (2). Although the pathogenesis of autoimmune diseases is still unclear, numerous studies have shown that aberrant innate and adaptive immunity is involved in the pathogenesis of autoimmune diseases (3, 4). Recently, emerging appreciation showed that NLRP3 inflammasome plays an important role in recognizing innate immune signals and inducing autoreactive immune responses, which probably acts as a checkpoint in innate immunity to cause skewed adaptive immune responses (**Figure 1**).

**Figure 2:**
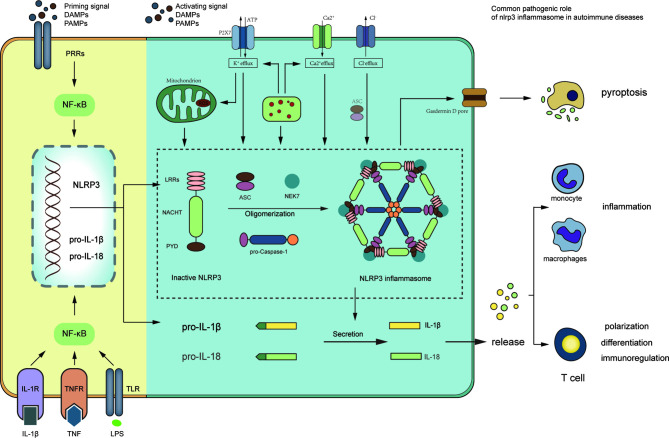
Signaling pathway of NLRP3 inflammasome and the role of NLRP3 inflammasome in autoimmune diseases. The priming stage: the binding of cytokines or PAMPs to its receptors can activate NF-κB signaling pathway, which upregulates transcription of NLRP3, pro-IL-1β, and pro-IL-18. The activation stage: this stage is stimulated by PAMPs and DAMPs. The activating signals, including K^+^ efflux, Ca^2+^flux, Cl^−^ efflux, mitochondrial dysfunction, ROS production, lysosomal disruption, and NEK7 promote oligomerization of NLRP3, ASC, and pro-caspase-1 to format NLRP3 inflammasome complex. The active caspase-1 can cleave proinflammatory cytokines IL-1β and IL-18, and promote pyroptosis mediated by Gasdermin D. The role of NLRP3 inflammasome in autoimmune diseases: inflammation promoted by cytokines IL-1β and IL-18; adaptive immune dysfunction caused by cytokines, such as proliferation and differentiation of T cells; pyroptosis, which can cause histiocytic death such as insulin β-cells.

Inflammasomes are protein complexes composed of three parts: a sensor, an adaptor, and an effector. Tschopp et al. firstly proposed the concept of inflammasomes in 2002 (5), following which several inflammasome subtypes were discovered, including NLRP1 inflammasome, NLRP3 inflammasome, absent in melanoma 2 (AIM2) inflammasome, etc. In most of the inflammasome subtypes, the component of adaptor is usually apoptosis-associated speck-like protein (ASC), which contains a caspase activation and recruitment domain. The effector component is usually caspase-1. The differences among inflammasomes subtypes are the sensor component (**Figure 2**).

NOD-like receptor (NLR) is a typical type of pattern recognition receptors (PRRs), *via* which innate immune system recognizes pathogen-associated molecular patterns (PAMPs) and damage-associated molecular patterns (DAMPs). According to different subcellular localization, PRRs are classified into two categories: (a) PRRs located in the plasma membrane, which mainly play the role to recognize PAMPs and DAMPs, typically including Toll-like receptors (TLRs) and C-type lectin receptors (CLRs); (b) PRRs located in intracellular partitions, which mainly include NLRs, AIM2-like receptor (ALRs), and cytosolic sensor cyclic GMP-AMP (cGAMP) synthase (6, 7). NLRP3 inflammasome is one of the most widely studied inflammasomes. Effect of NLRP3 inflammasome to various physiological and pathogenic stimuli mainly includes caspase-1 activation, secretion of IL-1β and IL-18, and pyroptosis mediated by caspase-1. Under physiological conditions, inflammasomes play an important role in clearing pathogens and damaged cells, and serve as a critical composition of innate immune response. Whereas under pathological conditions, the overactivation of inflammasomes may trigger autoinflammatory and autoimmune responses and result in numerous diseases.

In this review, we summarized the references and presented that as the checkpoint, NLRP3 inflammasome connects innate and adaptive immunity in several autoimmune diseases, including inflammatory bowel disease, psoriasis, rheumatoid arthritis, systemic sclerosis, type 1 diabetes, systemic lupus erythematosus, and autoimmune thyroid diseases. Finally, we discussed the effect of new-onset inhibitors of NLRP3 inflammasome in autoimmune diseases, which implies their potential therapeutic value for clinical applications deserved further study.

## Structure, Activation, and Regulation of NLRP3 Inflammasome

### Structure

NLRP3 inflammasome consists of three components including NLRP3 scaffold, a pyrin domain (PYD), and a caspase recruitment domain (CARD), known as ASC, and caspase-1 (8). NLRP3 belongs to NLR protein family which includes 22 members widely expressed in human histiocytes, such as dendritic cells, macrophages, and monocytes. NLRP3 contains three segments (**Figure 2**): (a) PYD in amino-terminal; (b) a NACHT domain in central part: executes the function of NLRP3 self-association *via* ATPase activity; (c) a leucine-rich repeat domain (LRR domain) in carboxy-terminal: depresses NLRP3 activation by inhibiting the ATPase activity of the NACHT domain. ASC includes PYD in amino-terminal and CARD in carboxy-terminal, which interact with each other to activate caspase-1. Caspase-1 consists of three parts from amino-terminal to carboxy-terminal: CARD, central large catalytic domain (p20), and small catalytic subunit domain (p10) (9, 10).

All of the substructures execute specific function in NLRP3 inflammasome assembly. Activated by upstream signals, the NACHT domains of NLRP3 interact with each other to induce NLRP3 oligomerization. And then the homotypic PYD-PYD interaction promotes ASC recruitments and formation of nucleates helical ASC filament. ASC recruits and activates caspase-1 *via* homotypic CARD-CARD interactions. At last, the clustered caspase-1 cleaves to a complex of p33 which comprises CARD and p20, a formation with proteolytical activation (11, **Figure 2**). NIMA-related kinase 7 (NEK7) is a new component in recent studies (**Figure 2**). Genetic research showed that NEK7 functioned as an indispensable component to NLRP3 inflammasome activation (12). NEK7 belongs to the family of mammalian NIMA-related kinases and is a serine or threonine kinase involved in mitosis. In the mediation of potassium efflux and mitochondrial reactive oxygen species (ROS), NEK7 binds to LRR domain of NLRP3, which is essential to NLRP3 inflammasome activation (13).

### Activation

The activation of NLRP3 inflammasome includes two steps: firstly, it should be primed, sequentially activated. Priming is a preparation stage for subsequent responses. On the one hand, upregulated expression of NLRP3, caspase-1, and pro-IL-1β is induced by gene transcription and activation of nuclear factor-κB (NF-κB). This process is initiated through three ways (**Figure 2**): (a) PRRs, such as NOD2 or TLRs, recognize PAMPs and DAMPs; (b) cytokines directly activate NF-κB pathway, including tumor necrosis factor (TNF) and IL-1β; (c) lipopolysaccharide (LPS) upregulates IL-1β transcription by shifting specific metabolism status (14–16). On the other hand, NLRP3 post-translational modifications (PTMs) are induced in the priming stage. NLRP3 is stabilized into a state in which the NLRP3 activity is auto-suppressed, but it still can respond to various signals (17). Importantly, NLRP3 PTMs occurs in the whole process of NLRP3 inflammasome activation, even in the unstimulated stage (18).

PAMPs and DAMPs stimuli varies in chemical properties and structures, and the direct binding of stimuli to NLRP3 are rarely detected. It is hypothesized that NLRP3 may sense common upstream signals which are induced by NLRP3 activators. This character of indirect activation is the core to understand molecular mechanism of NLRP3 activation. The comprehensive signal has not been determined, and relevant researches are contradictory. The common upstream signals identified include K^+^ efflux, Ca^2+^ flux, Cl^−^ efflux, mitochondrial dysfunction, ROS production, lysosomal damage, trans-Golgi disassembly, metabolic changes, and so on. There are three typical consensus models of common upstream signals.

#### Model of Ion Fluxes

The ion fluxes are common triggers of NLRP3 inflammasome activation. They include K^+^ efflux, Ca^2+^ flux, and Cl^−^ efflux, of which K^+^ efflux is an indispensable upstream event. It was first proposed that ATP-mediated P2X purinoceptor 7 (P2X7) promoted production of mature IL-1β through K^+^ efflux (19). And the high concentration of extracellular K^+^ can depress NLRP3 inflammasome activation (20, 21). It suggests that intracellular low potassium status may trigger NLRP3 inflammasome activation. Meanwhile, the activated P2X7-ATP-dependent pore recruits a pannexin-1 hemi-channel, through which extracellular agonists could enter the cytoplasm and interact with NLRP3 inflammasome complex to promote mature IL-1β secretion (22, 23). In addition, the silica, particulate stimuli alum and calcium pyrophosphate crystals are demonstrated to induce K^+^ efflux to trigger NLRP3 inflammasome activation (24). However, the mechanism of how intracellular decreased K^+^ levels trigger NLRP3 activation has not been precisely illuminated. One of the presuppositions suggested that the drop in intracellular K^+^ concentration may induce the conformational changes of NLRP3 to activate the following response (25, 26).

#### Model of Lysosomal Disruption

Particulate activation is a major factor to effect NLRP3 inflammasome. Self-derived particulate matter (such as uric acid, cholesterol crystals) and foreign-derived particulate matter (such as asbestos, alum, silica) could be endocytosed by lysosomal and cause subsequent lysosome membrane damages and release of the particulates and cathepsin B (27). NLRP3 inflammasome can be suppressed by broad-spectrum cathepsin inhibitors, such as CA-074-Me (a chemical cathepsin B inhibitor), which indicates that cathepsin is an important triggered signal of NLRP3 inflammasome (28, 29). Moreover, a variety of cathepsins exhibit function of promoting NLRP3 priming and activation, which is also blocked by CA-074-Me, and the NLRP3 activation process is rarely inhibited by the treatment of cathepsin B, X, L, or S gene deletion respectively (30). Hence, the redundancy among various cathepsin is vital for NLRP3 activation. In addition, lysosomal rupture can activate ion fluxes, including K^+^ efflux and Ca^2+^ influx. It indicates that ion fluxes may be a common-converged point in different NLRP3 activation models (25, **Figure 2**).

#### Model of Mitochondrial Dysfunction and ROS

The activated NLRP3 inflammasome can eliminate dysfunctional mitochondria and reduce ROS, which are vital upstream events in NLRP3 inflammasome activation. NLRP3 inflammasome activation can be blocked by ROS scavenging agents or NAPDH oxidase inhibitors (31). NLRP3 inflammasome activation was also detected in mouse macrophages and human peripheral blood monocytes which are depleted of NAPDH oxidase activity (32). Further study is required to determine the comprehensive role of mitochondria in NLRP3 inflammasome activation. In addition, mitochondria can provide a docking site for assembly of NLRP3 inflammasome. Cardiolipin, mitochondrial antiviral signaling protein, and mitofusin 2 may serve as the connective point of NLRP3 to the mitochondria (33, 34).

The priming and activation stages result in a multimeric protein complex assembled by NLRP3, ASC and caspase-1. There are two major functions of activated caspase-1: (a) Promoting maturity and release of IL-1β and IL-18 by cleaving their precursors; (b) Initiating pyroptosis, a specific cell death between necrosis and apoptosis, by cleaving gasdermin D (11, 35). IL-1β and IL-18 are important members of the IL-1 family, which also include IL-1α, IL-33, IL 37, and so on. The IL-1 family play vital roles in inflammatory responses and immune regulation (36). IL-1β, as a typical pro-inflammatory cytokine, can induce autoinflammatory response and tissue destruction. It can improve macrophages’ functions, recruit leukocytes *via* upregulating adhesion molecules and chemokines, and promote leukocytes to produce proinflammatory mediators (36–38). IL-1β, as a kind of T cell co-stimulatory factor, can provide pro-survival and proliferation signals for T cells (36), which also induce differentiation and polarization of T cell. IL-1β can promote naive CD4^+^ T cells differentiation into Th17 cells and Th9 cells cooperating with other cytokines (39). IL-18, as a kind of IFN γ-inducing factor, can induce natural killer (NK) cells to product IFN-γ and IL-8 (40). IL-18 induces Th2 responses and promotes Th1 responses, synergizing with which induce IFN-γ production of T helper cells (36, 41). In epithelial cells, IL-18 regulates function of Th17 cell and Treg cell, which contributes to Th17/Treg imbalance (42).

### Regulation

By recognizing pathological infections and endogenous danger signals, NLRP3 inflammasome can trigger immune responses and inflammatory responses. The regulation of NLRP3 inflammasome activation is as critical for immune regulation in immune homeostasis, as for inflammose function itself. The aberrant regulation can induce NLRP3 inflammasome overactivation and excessive cytokine production, resulting in skewed inflammatory responses, which might induce adaptive immune dysfunction. The statue can be switched from physiological defense to pathogenic damage by aberrant regulation.

The strict regulation of NLRP3 inflammasome activation is essential to maintain immune system homeostasis. More importantly, accurate understanding of the regulatory mechanisms is crucial to identify triggers of autoimmune diseases involved in NLRP3 inflammasome, and treatments targeted on it may show great therapeutic potential. Various potential regulation mechanisms have been reported, such as cell surface associated mucin 1, MicroRNA (miRNA), small heterodimer partner (**Table 1**). Clarifying traits of regulations from the following aspects might contribute to a more comprehensive understanding: positive or negative regulation; the specific stage on which the regulator targets, including priming stage and activation stage; and specific subcellular location, such as mitochondria and Golgi apparatus (**Table 1**).

**Table 1:** Regulation of NLRP3 inflammasome.

Regulation	Effect on NLRP3 inflammasome activation		Stage		Subcellular location
	Promote	Inhibit	Priming stage	Activation stage	
Cell surface associated mucin 1 depresses phosphorylation of interleukin 1 receptor associated kinase 1 to downregulate activity of NF-kB (43).		√	√		Nucleus
A20 depresses degradation of NF-kB essential modulator by ubiquitination (44).		√	√		Nucleus
2,3,7,8-tetrachlorodibenzo-P-dioxin mediate aryl hydrocarbon receptor to enter the cell nucleus and combine to ARNT, which could bind to xenobiotic response element (45).		√	√		Nucleus
MicroRNA (miRNA) targets on specific region of NLRP3 mRNA to inhibit its expression (46).		√	√		Nucleus
Ubiquitylation of NLRP3, a type of post-translational modifications (PTMs) of NLRP3 (18).	√		√	√	
Proteins bind to PYCARD competitively, such as POPs (PYD-only proteins), COPs (CARD-only proteins), PYNOD (NLRPIO), and so on (47).		√		√	Cytoplasm
Leucine-rich repeat flightless-interacting protein recruits lightless I (the inhibitor protein of caspase substrate) (48).		√		√	Cytoplasm
Small heterodimer partner combines to NLRP3 competed with ASC (49).		√		√	Mitochondria
Cellular Fas-associated death domain-like IL-1β converting enzyme inhibitory protein interact with NLRP3 and pro-caspase-1 (50).	√			√	Cytoplasm
The functions of mitochondria, such as providing a docking site for NLRP3 inflammasome assembly (51, 52).	√			√	Mitochondria
Lysosomal disruption induced by phagocytosis of particulates (28).	√			√	Lysosome and cytoplasm
The trans-Golgi network is disassembled into vesicles, which recruit NLRP3 and promote NLRP3 aggregation (53).	√			√	Golgi

PTMs of NLRP3 can participate in various phases of NLRP3 inflammasome activation and regulate innate immunity. Covalent additions including ubiquitylation, phosphorylation, and sumoylation have been reported in NLRP3 PTMs. For example, sumoylation by the protein E3 SUMO protein ligase MUL1 (also known as MAPL) depresses NLRP3 activation. However, both promoted and suppressed effect of phosphorylation on NLRP3 have been detected (54, 55). In addition, PTMs can interact with each other. Phosphorylation can promote deubiquitylation by interaction between BRCA1/BRCA2-containing complex subunit 3 and LRR domain of NLRP3 (54). It indicates that regulation of NLRP3 activation is a delicate and complex process.

Overreaction of innate immune responses may cause overactivity of cytokines and pyroptosis, which contribute to inflammatory and autoimmune diseases, the same as overreaction of skewed adaptive immune responses may cause hypersensitivity. Therefore, identifying critical triggers of the unbalanced immunoregulation would be an important direction in the future researches.

## NLRP3 Inflammasome in Autoimmune Diseases

Autoimmune diseases are characterized by loss of immunological tolerance and inappropriately autoreactive immune responses against histocytes and organs. However, the exact pathogenesis of autoimmune diseases has not been identified, and existing treatments are unsatisfactory (56). Current studies have proposed the role of NLRP3 inflammasome in autoimmune diseases and its clinical therapeutic potential (**Table 2**).

**Table 2:** Studies of the roles of NLRP3 inflammasome in autoimmune diseases.

	Effect of NLRP3 inflammasome		Regulation of NLRP3 inflammasome activation and relevant pathway in autoimmune diseases.	
	Specific mechanism	Common mechanism	Positive regulation	Negative regulation
IBD	Disrupted inflammasome responses result in dysbiosis and increased colonization of pathobionts (57). .	Physiological condition: sense and respond to foreign milieu in the extracellular environment, *via* pathogen-associated molecular patterns (PAMPs) and damage-associated molecular patterns (DAMPs), mediate host immune responses to microbial infection and cellular damage. Pathological conditions: 1) Histiocytic and organic inflammation promoted by cytokine IL-1 and IL-18; 2) Induce adaptive immune dysfunction *via* NLRP3 inflammasome activation, inducing the migration and differentiation of T cell by cytokines; 3) Pyroptosis modulated by activated caspase-1 of immunocyte and specific histocytes.	Transporter 4 (58); Oxazolone (59); Protein tyrosine phosphatase non-receptor 22 (60).	Fasting-mimicking diet (61); Nutrient deprivation (62); CircRNA HECTD1 (63); Naringin (64); Carboxyamidotriazole (65); Growth differentiation factor 11 (66); Phloretin (67); Nuclear factor E2-related factor-2 (68); Cardamonin (69); sDR5-Fc fusion protein (70); Hydrogen sulfide (71); Cinnamaldehyde (72); BBG (a P2X7R blocker) and OLT1177 (73).
Psoriasis	IL-1 participates in pathogenesis partially (74).		CD100/PlxnB2 (75); Tristetraprolin (TTP) downregulation (76); Tumor necrosis factor (TNF)-α (77); Acute-phase protein serum amyloid A (78).	Bay11-7082 (79); Datura Metel L (80).; Cycloastragenol (81); Cas9 RNP nanocomplexes (82).
RA	NLRP3 inflammasome activation contributes to Th1 differentiation in CD4^+^ T cells (83); Induce Th2 differentiation and antibodies production (84).		TNF-α and calreticulin (85); Calcium-sensing receptor (86); Tofacitinib (87).	MCC950 (88); Protectin DX (89); Taraxasterol (90); Celastrol (91); Punicalagin (92); Hsa_circ_0044235 (93); hUCB-MSCs (94); A20 (44); tristetraprolin (95).
SSC	Downstream factors including IL-1, IL-18, and miR15 promote collagen synthesis and fibrosis (96, 97).		MiR-155 (97); Parvovirus B19 (98).	
T1DM	IL-1β induces the migration of proinflammatory cells into pancreatic islets (99, 100); IL-1β has direct cytotoxic effects on beta-cells (99, 100); Autoreactive T cells infiltrate pancreatic islets and cause beta-cell death (101).		LPS+ATP (102); Nitric oxide (103); Metabolic stress (104); Mitochondrial DNA (mDNA) (105).	Verapamil (106); Scutellarin (107); Ginsenoside Rg1 (108); Low-methoxyl pectin (109); fingolimod (110).
SLE	Autoantibodies induce NLRP3 inflammasome activation (82, 111); accumulation of NETs contributes to the pathogenesis (112, 113).		U1-small nuclear ribonucleoprotein (114); Glycogen synthase kinase 3β (115); Cyclic GMP-AMP synthase (116); Neutrophil extracellular traps (NETs) (117); Surface CXCR2 expression (118); Reactive oxygen species (119).	Xenon (120); Honokiol (121); Tris (dibenzylideneacetone) dipalladium (122); Cf-02 (123); Let-7f-5p (124); Magnolol Bay11-7082 (125); Curcumin (126); Melatonin (127); Lcariin (128); Piperine (129); Citral (130).
AITDs			Excessive iodine (131).	Yanghe decoction (132).

### Inflammatory Bowel Disease

Inflammatory bowel disease (IBD) is the most common intestinal tract disorders, characterized by dysfunction of innate immunity and aberrant inflammation in intestinal mucosa. It is comprised mainly of Crohn’s disease (CD) and ulcerative colitis (UC) (133). Though the specific pathogenesis of IBD has not been elucidated until now, several researches have revealed that NLRP3 inflammasome activation is upregulated in IBD. Serum concentration of NLRP3 is elevated, positively correlated with serum IL-1β level and severity of IBD patients (134). Furtherly, NLRP3 inflammasome is activated in early stage of CD patients, whereas in late stage of UC patients, which implies that there are differences between the development of UC and DC (135).

Downstream effects of NLRP3 inflammasome activation contribute to chronic inflammation, alterations in innate immune responses and disorders in mucosal immune response. These effects mainly include the consequent processes: pro-inflammatory cytokine release, macrophage hyperactivation with uncontrolled damage, and pyroptosis (136). *In vitro*, Monocarboxylate Transporter 4 contributed to intestinal enterocyte pyroptosis mediated by Caspase-1 through ERK1/2-NF-κB pathway, which can promote intestinal inflammation (58). Recent studies show that non-canonical endogenous irritants such as non-infectious stress conditions can also affect NLRP3 inflammasome activation. Fasting-mimicking diet reduced the expression of NLRP3 inflammasome and CD4^+^ T cells percentage in peripheral blood and spleen to alleviate intestinal inflammation, which indicated that calorie restriction might modulate immune response *via* NLRP3 inflammasome (61). Yun et al. presented that NLRP3 inflammasome was removed by ubiquitin-mediated degradation, which was promoted by autophagy under nutrient deprivation (62). The downstream proinflammatory cytokines IL-1and IL-17E/25 could be suppressed to strengthen intestinal barrier function (62). CircRNA HECTD1 (circHECTD1) can alleviate UC by inhibiting NLRP3 inflammasome. And in Caco-2 cells, circHECTD1 can induce human antigen R (HuR) *via* miR-182-5p, which contributes to NLRP3 inflammasome activation by autophagy (63). NLRP3 inflammasome may have a double effect on IBD: some reports showed that IL-1β and IL-18 play a protective role in gastrointestinal inflammation. Oxazolone can stimulate maturation of pro-caspase-1 and pro-IL-1β, and colitis induced by oxazolone can be ameliorated by exogenous IL-1β or IL-18 (59). Higher sensitivity to oxazolone treatment and decreased IL-1β or IL-18 production were detected in NLRP3^(−/−)^ mice compared to wild-type mice, which indicates that IL-1β and IL-18 derived from NLRP3 inflammasome can protect against inflammation in gastrointestinal mucosa (59). In addition, IL-1β contributes to enhanced host defense against *Clostridium difficile* and *Citrobacter rodentium* during acute affection (137, 138). It induces ASC-dependent CXCL1 production to recruit neutrophils to the intestine, protects epithelial integrity, and reduces colonization (137, 138). There are several explanations for the protective effect. Marianne et al. presented that protein tyrosine phosphatase non-receptor 22 mediates dephosphorylation of NLRP3 inflammasome, resulting in its activation and release of mature IL-1β in mouse models (60). Under a physiological condition, this process contributes to effective host defense against harmful pathogens leading to gastrointestinal disorder and subsequent reconstitution (60). Meanwhile, another study had shown that NLRP3 inflammasome could promote neutrophil chemotaxis and antimicrobial secretions of the colon to sustain intestinal homeostasis (57). When epithelial barrier was destroyed, microbiota recruited immune cells to the lamina propria, and NLRP3 inflammasome activated in these immune cells played destructive effect (57). Therefore, NLRP3 inflammasome may have different effects in specific conditions, including physiological condition, infection by invading pathogenic bacteria and involving in innate immune disorder.

NLRP3 inflammasome inhibitors have been demonstrated to be effective in experimental models of IBD. Most inhibitors have a commonly downregulated effect on NLRP3, caspase-1, and pro-inflammatory cytokines. So far, therapeutic targets of NF-κB, Nuclear factor E2-related factor-2 (Nrf2), and ROS have been widely studied. Naringin, an activating ligand of peroxisome proliferator-activated receptor γ (PPARγ), can significantly decrease disease activity indexes (DAI), pathological damage of colon, and inflammation severity (64). Naringin alleviated UC *via* pressing NLRP3 inflammasome by activating PPARγ and degrading subsequent NF-κB activation (64). In a murine TNBS-induced colitis model, carboxyamidotriazolez downregulated NF-κB pathway by reduction of NF-κB p65 expression and phosphorylation of IκBα (65). Phloretin and Growth differentiation factor 11 play an anti-inflammatory effect targeting on the same pathway (66, 67). Nrf2 is a negative regulator for NLRP3 inflammasome by inhibiting priming of NLRP3 inflammasome (68). Cardamonin is a natural herbal extract of Alpinia katsumadai Hayata. Nrf2 and its target genes NQO1, Trx1, SOD2 and HO-1, especially NQO1, was elevated by cardamonin, which has the effect to depress NLRP3 inflammasome activation in a mouse model (69). And upregulation of Nrf2 has been detected after treatment of Resveratrol and Hydrogen sulfide (H_2_S) (70, 71). ROS is considered as a second messenger in pro-inflammatory responses. H_2_S can also reduce ROS production to prevent inflammation (71). Moreover, therapeutic strategies focused on miRNA has attracted more attention, Cinnamaldehyde can decrease MicroRNA-21 and miR-155 levels in colons and macrophages to ameliorate DSS-induced colitis (72). Recent studies showed that new mode of administration can also promote the efficacy of NLRP3 inflammasome inhibitors. The combined administration of BBG (a P2X7R blocker) and OLT1177 (a selective NLRP3 inhibitor) effectively alleviates UC by complementary effects (73). The effect of NLRP3 inflammasome inhibitors in IBD has not been comprehensively understood, which deserves further study.

### Psoriasis

Psoriasis is a chronic inflammatory skin disease mediated by immune responses. In psoriasis biopsy, the expression of NLRP3, caspase-1, and IL-1β was significantly upregulated compared to non-lesional psoriatic skin (139). The single nucleotide polymorphisms (SNPs) studies show that the genetic mutations in NLRP3 are associated with psoriasis susceptibility in Chinese Han population (140). And the genetic mutations in *CARD8-C10X* (*rs2043211*) are associated with Psoriatic arthritis (PsA) in northern Swedish population (141). All these findings indicate that NLRP3 inflammasome may be involved in the occurrence and development of psoriasis.

PlxnB2 and its ligand (such as CD100) participate in neuronal development and immune responses (75). The bound of soluble CD100 to PlxnB2 can upregulate NF-κB pathway, which induces NLRP3 inflammasome activation in keratinocytes of psoriasis patients (75). In fibroblasts deriving from patients with psoriasis, abnormal inflammasome activity can be induced by downregulation of tristetraprolin TTP (76). TTP can directly target the degradation of NLRP3 mRNA; therefore, TTP downregulation may contribute to pathogenesis of psoriasis *via* NLRP3 inflammasome activation (76). Verma et al. found that TNF-α could activate NLRP3 inflammasome, which is independent of priming signals, and caspase-1 reactivity, plasma IL-1β and IL-18 are reduced in psoriasis patients treated with anti-TNF therapy (77). In mouse model of psoriasis, NLRP3 inflammasome keratinocytes can be activated by TNF-α to induce inflammation, which was induced by inhibiting autophagy *via* PI3K/AKT/mTOR signaling pathway (142). Overexpression of lncRNA MEG3 could alleviate inflammation by promoting autophagy (142). Serum amyloid A (SAA) serves as an important trigger to promote expression of IL-1β by activating NF-κB pathway in psoriatic keratinocytes (78). And IL-1β plays a key role in the skin diseases mediated by T helper type 17 cells (78). *In vitro*, miR-155 silencing can depress NLRP3/caspase-1 signal pathway to alleviate inflammatory responses (79). In summary, these findings indicate that CD100/PlxnB2, TTP, TNF, miR-155, and SAA might be potential therapeutic targets for psoriasis.

Herbal extracts have been demonstrated to be effective treatment for psoriasis. In psoriasis mouse models induced by imiquimod, the expression of TLR7, TLR8, p-NF-κB, and NLRP3 were obviously suppressed by administration of Datura metel L (80). The production of key inflammatory cytokines including IL-1β, IL-17, and IL-23 was also inhibited, which implied that Datura metel L. plays a protective role by depressing the pathway of TLR7/8-MyD88-NF-κb-NLRP3 (80). Paeonia lactiflora Pallas extract and BAY11-7082 have an inhibitory effect against immune responses in keratinocytes probably by the similar pathway (79, 81). In addition, cycloastragenol specifically inhibits macrophages infiltration in dermis and pyroptosis mediated by NLRP3 inflammasome in mouse models (81). IL-1 inhibition might be another promising therapeutic strategy. Wan et al. reported a dissolvable microneedle, which consists of Cas9 RNP nanocomplexes and dexamethasone nanoparticles (82). The microneedle can be internalized by keratinocytes and immune cells, and disrupted NLRP3 inflammasome selectively to alleviate skin inflammations in a mouse model of psoriasis (82). Of note, new-onset plaque psoriasis has been shown to be a side effect in the RA patients treated with anti-IL-1 therapy (74). The role of NLRP3 inflammasome in psoriasis pathogenic mechanism is complicated, and the researches are comparatively limited, which deserves further studies.

### Rheumatoid Arthritis

Rheumatoid arthritis (RA) is a common chronic autoimmune disease characterized by persistent synovial inflammation, pannus formation, destruction of cartilage and small diarthrodial joints, which is mainly caused by autoantibody secretion and aberrant immune responses (143). Various NLRP3-releated SNPs have been shown to be associated with susceptibility of RA. NLRP3 SNPs are associated with susceptibility of RA and anti-TNF responses in Caucasian population (144). In addition, genetic mutations of NLRP3 inflammasome may increase the risk of stroke and TIA in Swedish population, but not of myocardial infarction and angina pectoris (145).

Several studies have shown the hyperactivity of NLRP3 Inflammasome and downstream factors in RA. The intracellular levels of NLRP3, active caspase-1, pro-IL-1β, and active IL-1β in whole blood cells are increased in active RA patients (146). This increased level can also be detected with the treatment of TLR4 or TLR3 agonist (146). Guo et al. found that MCC950, a selective NLRP3 inhibitor, is able to depress NLRP3 inflammasome activation in monocyte and macrophages to infiltrate into the synovia, resulting in lesser joint inflammation and bone destruction (88). It indicates that NLRP3 Inflammasome is involved in the pathogenesis of RA. NLRP3 inflammasome activation has not been detected in fibroblast-like synoviocytes (FLS), which suggests that FLS may not produce IL-1β mediated by NLRP3 inflammation (147). TLR1-9 have been demonstrated to play a modulatory role in joint inflammation in the pathogenesis of RA (148). Downregulation of NOD2 gene expression can decrease NF-κB and pro-inflammatory cytokines in FLS of RA patients, which indicates that NOD2 may promote NLRP3 Inflammasome activation by effecting the priming stage (149). Both in FLS and human umbilical vein endothelial cells of RA patients, TNF-α/CRT dual signaling promotes NLRP3 Inflammasome activation *via* enhancing the effect of caspase-1 (85). TNF-α may serve as an initiator for HuR translocating to mediate NLRP3 Inflammasome activation (85). The typical activated models of Ca^2+^ and P2X7 are also detected to initiate activation of NLRP3 Inflammasome in RA patients (86).

Zhao et al. demonstrated that NLRP3 Inflammasome promotes Th17 cell differentiation to enhance the adaptive immune dysfunction of RA (150). Th17 has been proven to have a vital role in the downstream cascade reactions of NLRP3 inflammasome in RA. Jin et al. firstly found that protectin DX downregulates Th17 cells and pro-inflammatory cytokines and upregulates Tregs and anti-inflammatory cytokines by inhibiting NLRP3 inflammasome activation *via* miR-20a (89). Tofacitinib effectively ameliorates the severity of RA by restoring Treg/Th17 cell balance *via* reducing NLRP3 inflammasome activation in arthritic joints and draining lymph nodes (87). NLRP3 inflammasome can also mediate secretion of Fas-associated death domain, which is a pivotal adaptor molecule in innate immunity and inflammation (151). However, the expression of NLRP3 and pro-caspase-1 was decreased in the peripheral neutrophils (152). The active caspase-1 expression was increased, which was positively correlated with serum level of IL-18, which suggested that IL-18 mediated by active caspase-1 plays a pro-inflammatory role in neutrophils of RA, independently of NLRP3 inflammasome (152). Thus, different cell types should be noted in the pathogenesis of RA.

Taraxasterol exerts anti-inflammatory effects by depressing NLRP3 inflammasome pathway *via* downregulating the expression of NF-κB in RA patients (90). Celastrol, a quinone-methylated triterpenoid extracted from Tripterygium wilfordii, alleviates RA inflammation by suppressing the ROS/NF-κB/NLRP3 pathway (91). In collagen-induced arthritis mouse models, Punicalagin (an active substance extracted from pomegranate peel) inhibits phenotype polarization and pyroptosis of M1 macrophages by downregulating NF-κB signaling (92). Hsa_circ_0044235 has been found to inhibit NLRP3-mediated pyroptosis in FLSs (93). Other potential therapeutic strategies focus on negative regulation of NLRP3 inflammasome. Human umbilical cord blood-derived MSCs suppress the expression of NLRP3 Inflammasome *via* a paracrine loop of IL-1β signaling in CIA mouse models and mononuclear cells from RA patients (94). The deficiency of A20 in macrophages can enhance the effect of NLRP3 inflammasome by promoting the biological activity of caspase-1, IL-1β secretion, and pyroptosis in mouse models (44). Activation of NLRP3 Inflammasome and secretion of IL-1β can be inhibited by TTP expression and activation of TTP in both *in vivo* and *in vitro* models (95). TTP can be activated by dephosphorylation mediated by protein phosphatase 2A (PP2A), and Arctigenin (PP2A agonist) reduces monosodium urate (MSU) crystal-induced inflammation (95). It is indicated that TTP might be a potential target for inflammation induced by MSU crystals (95). These studies indicate that treatments aiming to inhibit NLRP3 inflammasome and IL-1β might be potential therapy for RA.

### Systemic Sclerosis

Systemic sclerosis (SSc) is an idiopathic autoimmune disease targeting on connective tissue, characterized by autoimmune dysfunction, microvascular vessel alterations, and skin fibrosis. The fibrosis is triggered by inflammatory response, in which innate immunity serves as a key factor. However, the precise pathogenesis hasn’t been clarified, and few effective treatments have been available until now.

In skin of patients with SSc, NLRP3 is upregulated and correlates positively with skin thickness (153). A resistant feature to skin fibrosis can be detected in NLRP3^(−/−)^ mice and ASC ^(−/−)^ mice, which implies that there is a correlativity between SNPs of NLRP3 and SSc (96). MiR-155 participates in innate immunity and adaptive immunity (154, 155). MiR-155 is involved in pulmonary fibrosis, hepatic fibrosis, as well as wound site fibrosis (156–158), and IL-1β can induce the expression of miR-155 (159). The expression of miR-155 was significantly increased in SSc lung fibroblasts, and fibrosis could be driven by inflammasome-dependent expression of miR-155 (97). Furthermore, there are positive feedbacks between NLRP3 inflammasome and miR-155, which explains sustaining fibrosis in SSc (97). MiR-155 might be a potential target for SSc, deserving future study.

Artlett et al. detected that caspase-1 inhibitor decreased secretion of IL-1β, IL-18, and collagens in dermal and lung fibroblasts of SSc, and reduced expression of α-smooth muscle actin in dermal fibroblasts (96). Shinohara presented that host receptors can identify pathogens to induce autoimmunity, which implies that infections and autoimmunity are closely connected (160). B19V infection induces caspase-1 mediated by NLRP3 inflammasome in monocytes of SSc patients (98). The expression of IL-1β and TNF-α can be induced by stimulus such as lipopolysaccharides (98). It indicates that viral components can enhance the sensitivity of NLRP3 inflammasome activation in monocytes (98). These findings may provide potential prevention strategy for SSc.

### Type 1 Diabetes

Type 1 diabetes (T1D) is an autoimmune disease targeting insulin-producing pancreatic β-cells specifically mediated by T lymphocyte (161). Both innate immunity and adaptive immunity participate in the development of T1D, and NLRP3 inflammasome acts as an important component of innate immunity to induce insulitis and β-cell death (162, 163). Polymorphisms of NLRP3 inflammasome-related gene correlate with T1D. Pontillo et al. found that SNPs in NLRP3 had a correlation specifically with T1D in Brazilian population (164). In Norwegian population and Chinese Han population, NLRP1 was associated strongly with T1DM (165). Wu et al. found that SNPs in NLRP3 correlated specifically with T1D, especially in Latin American population (166). In Slovenian population, the association of *NLRP3* polymorphisms with T1D has not been observed (167). It is needed to confirm the specific gene polymorphisms of NLRP3 in different populations.

The important role of IL-1β in the pathogenesis of T1D has been determined in previous studies. IL-1β modulates pro-inflammatory cells to migrate into pancreatic islet, inducing direct cytotoxicity and β-cell apoptosis, which depends on the dose of IL-1β in T1D rat models (99, 100). Interestingly, NLRP3 inflammasome played a protective role in early stage of T1D in IRAK-M ^(−/−)^ NOD mice (168). In human islets, NLRP3 inflammasome can be activated, and secretion of IL-1β increases by the presence of LPS and ATP (102). As an upstream factor activating IL-1β, NLRP3 inflammasome may have a potential function in T1D. Resident peritoneal macrophages promote inflammation by inducing inflammatory cytokine/chemokine secretion such as IL-1β and upregulating the receptor expression of these proinflammatory cytokine/chemokine (103). Nitric oxide production is mediated by upregulated NLRP3/iNOS (nitric oxide synthase) pathway, which contributes to the proinflammatory state of resident PMs (103).

NLRP3 inflammasome has been shown to initiate and participate chronic complications of diabetes. Hyperglycemia can activate NLRP3 inflammasome, promote secretion of inflammatory cytokines, and trigger cascade of inflammatory response, finally inducing diabetic nephropathy, diabetic retinopathy, and so on (104, 169). Mitochondrial DNA activates NLRP3 inflammasome in endothelial cells *via* Ca^2+^ influx and mitochondrial ROS generation, which mediates endothelial dysfunction and vascular inflammation in diabetes complications (105). NLRP3 inflammasome activation can be detected in urothelial cells (170). It induces loss of in nerve density and Aδ-fibers, and leading to insensitivity of bladder fullness, which is a specific diabetic bladder dysfunction symptom (170). Verapamil, a calcium channel blocker, can diminish the release of IL-1β and TNF-α into the vitreous fluid and decrease retinal ganglion cell loss in diabetic retinopathy *via* inhibiting NLRP3 inflammasome mediated by TLR4 (106). Verapamil can also reduce pancreatic islets shrinkage and enhanced CD34 expression by interfering NLRP3 inflammasome assembly (106). In streptozotocin-induced mice with diabetic cardiomyopathy, the expression of NLRP3 inflammasome, IL-1β, and IL-18 in cardiac tissues was induced by nuclear NF-κB translocation, which can be inhibited by Scutellarin treatment (107). These researches imply that NLRP3 inhibitors could serve as a potential target for patients with diabetes.

MCC950, as an inhibitor of NLRP3 inflammasome, has been demonstrated to be a compelling treatment for diabetes in mouse models (171). Gao et al. found that Ginsenoside Rg1, a major active ingredient in ginseng, a traditional herb for diabetes in China, had a function to weaken NLRP3 activity in the liver and pancreas (108). In addition, low-methoxyl pectin can mediate decrease of NLRP3 inflammasome activation. And it can enhance cecal barrier function and shape intestinal homeostasis to ameliorate gut-pancreatic immune environment (109, 172). Low-methoxyl pectin may serve as a promising prevention drug of T1D. The TLR2/4, NF-κB, and NLRP3 inflammasome pathways are upregulated in intestinal tissues of NOD mice, which promotes the secretion of downstream signaling proteins such as IL-1β and IFN-γ (110). These downstream signaling proteins can induce activation and differentiation of T cells and the migration of these diabetogenic T cells to the pancreas (110). We propose that immunotherapy targeting on NLRP3 inflammasome is a promising approach to treat T1D.

### Systemic Lupus Erythematosus

Systemic lupus erythematosus (SLE) is a systemic autoimmune disease characterized by production of autoantibodies against nuclear components, deposition of immune complex, and multiorgan damage resulting from aberrations of immune response. Gene polymorphisms of NLRP3 were significantly associated with susceptibility of SLE in Latin American individuals (173). However, in Chinese Han population, association between NLRP3 SNPs and SLE susceptibility has not been observed (174). It is warranted to study the relationship of gene polymorphisms of NLRP3 with SLE susceptibility in different populations. The expression of NLRP3 inflammasome, AIM2, and caspase-1 was increased in renal tissues, especially there was a positive relationship between the expression level of NLRP3 inflammasome and the activity index score in patients with SLE (175). Furthermore, the expression of NLRP3 mRNA was upregulated in macrophages, and the expression of AIM2 mRNA was decreased in female SLE patients (176). Meanwhile, in male SLE patients, the expression of AIM2 mRNA was increased, and SNP of CARD8 resulted in susceptibility of patients (176). It implied that there is a gender-dependent difference in mechanism of inflammasome activation in SLE patients.

Both innate and adaptive immunity are involved in the pathogenesis of SLE. Double-strand DNA and immune complex could activate NLRP3 inflammasome in histiocytes to promote inflammatory response mediated by IL-1β and IL-18 (49). Nucleic acid components including microbial nucleic acids, endogenous DNA, and endogenous RNA-containing U1-small nuclear ribonucleoprotein (U1-snRNP) can activate NLRP3 inflammasome in human monocytes *in vitro* (114). Shin MS et al. discovered that immune complexes could activate NLRP3 inflammasome by upregulating NF-kB pathway in animal models and patients with SLE (111). The caspase-1 activation and IL-1β production of bone marrow-derived macrophages was decreased after it was transfected with Glycogen synthase kinase 3β (GSK-3β) siRNA (115). The administration of GSK-3β inhibitor could alleviate severe proteinuria and nephritis, and anti-dsDNA antibody production, deposition of immune complex, and circulating cytokines were depressed (115). Zhao et al. suggested that GSK-3 promotes renal inflammation by activating NLRP3 inflammasome and mediating IL-1β release (115). Caspase-1 was activated especially in CD14-positive and CD16-positive monocytes from SLE patients, which were positively correlated with serum titers of anti-double-stranded DNA antibodies and negatively correlated with serum levels of complement component 3 and platelet count (116). NLRP3 inflammasome could be activated by cyclic GMP-AMP synthase stimulator of interferon genes pathway to promote caspase-1 activation and IL-1β secretion (116). Neutrophil extracellular traps (NETs) were also found to participate in the pathogenesis of SLE. NETs are a network consisting of chromatin fibers and granule-derived antimicrobial peptides (177). The impaired clearance of NETs and increased release of NETs are promoted by low-density granulocytes (LDGs), which contribute to accumulation of NETs in human monocytes *in vitro* (112, 113). NETs can activate caspase-1 with releasing of proinflammatory cytokines IL-1β and IL-18, and promote the formation of immune complex and type I interferon (117). Interestingly, IL-18 in turn induces perpetual NETosis in human neutrophils, which results in a feed-forward inflammatory loop (117). A recent study found that milk fat globule-EGF factor 8 (MFG-E8) could reduce neutrophil migration, accumulation, phagocytosis, and NETosis *via* reducing surface CXCR2 expression in pristane-induced lupus and patients with SLE (118).

NLRP3 inflammasome plays an important role in the development of SLE. Podocytes are highly differentiated epithelial cells and a critical component of glomerular basement membrane (GBM), which play a core role in maintaining the function of glomerular filtration (178). In the later stage of SLE, the expression of NLRP3 inflammasome and caspase-1 could be detected in podocytes in murine lupus models (115). The production of ROS promotes NLRP3 inflammasome activation in the podocytes line (119). Importantly, TLRs (such as TLR4) is expressed in glomerular podocytes of normal mice, which implies that podocytes also possess immune function in physiological condition (179). In addition, IL-18 release mediated by NLRP3 inflammasome is a potential pathogenic factor in cutaneous lupus lesions. Type I IFN is reported to be a main risk factor of cardiovascular disease (180). The feed-forward inflammatory loop mentioned above can also be detected in endothelial progenitor cells (181). Xenon can reduce NF-κB/NLRP3 inflammasome activation to ameliorating renal function in mouse models with spontaneous LN (120). Honokiol and dibenzylideneacetone (Tris) dipalladium both have potential therapeutic effect against accelerated and severe type of lupus nephritis by suppressing NLRP3 inflammasome activation (121, 122). The former interferes NLRP3 inflammasome *via* reducing NF-κB activation, suppressing reactive oxygen production and mitochondrial damage, and inducing sirtuin 1/autophagy axis activation (121). And the latter can reduce p38 MAPK signaling pathways and regulate the autophagy/NLRP3 inflammasome axis (122). Cf-02 can treat acute onset of severe lupus nephritis in mice by inhibiting the NF-κB/NLRP3 inflammasome axis and regulating T cell functions differentially (123).

Interestingly, NLRP3 inflammasome might have immunosuppressive effect on SLE (182–184). The expression of TGF-β target genes was depressed by deficiency of NLRP3 and ASC in mice models of spontaneous lupus-like autoimmunity (182). NLRP3 and ASC are demonstrated to downregulate TGF-β receptor signaling through SMAD2/3 phosphorylation, which contributes to the immunosuppressive effect (182). There are more and more new drugs targeting the NLRP3 inflammasome in the treatment for SLE, and some show to be effective (**Table 2**).

### Autoimmune Thyroid Diseases

Autoimmune thyroid diseases (AITDs) are a series of thyroid diseases characterized by thyroid tissue damage and autoimmune disorders, including mainly Hashimoto’s thyroiditis (HT) and Graves’ disease (GD).

Guo et al. proposed firstly that multiple inflammasomes including NLRP3, NLRP1, NLRC4, and AIM2 participated in the development of AITDs (185). Excessive iodine promoted pyroptosis activity in thyroid follicular cells *via* the ROS-NF-κB-NLRP3 pathway which might be involved in the development of HT (186). However, Nagayama, Y., raised questions that Nthy-ori 3-1 cells aren’t capable of iodine uptake and the concentration of iodine (131). The role of NLRP3 inflammasome in Graves’ disease (GD) has not been reported so far.

It is reported that Yanghe decoction (a traditional Chinese herbal formulation) can alleviate autoimmune thyroiditis in rat models *via* downregulating NLRP3 inflammasome and adjusting the imbalance Th17/Treg (132). There are very few studies concerning the effect of NLRP3 inflammasome in the pathogenesis of AITDs, deserving further study.

## The Possible Pathogenesis of NLRP3 Inflammasome in Autoimmune Diseases

NLRP3 inflammasome exists in various immune cells, including macrophages, granulocytes, antigen-presenting cells, B cells, and T cells. It has been found in tissues and cells such as platelet, podocyte, and keratinizing squamous epithelium of skin (187). As a vital component of innate immune system, NLRP3 inflammasome recognizes DAMPs and PAMPs, and initiates innate inflammatory responses *via* promoting proinflammatory cytokines secretion, which may also trigger adaptive immunity dysfunction (161). Besides its elementary physiological functions in immune responses, NLRP3 inflammasome also participates in pathogenesis process such as multiple autoimmune diseases. The common character of autoimmune diseases is histocyte and organ damage resulting from autoantibody overproduction, which leads to loss of immunological tolerance and aberrant autoreactive immune responses. The accurate role of NLRP3 inflammasome in autoimmune disease pathogenesis is complex and has not yet been completely illuminated.

Collectively, the effect of NLRP3 inflammasome in autoimmune diseases involves the following two aspects (**Table 2** and **Figure 2**): due to caspase-1 being the effector of inflammasome structure, IL-1β, IL-18, and pyroptosis modulated by activated caspase-1 play a major role in autoimmune diseases. Firstly, inflammation promoted by cytokines, especially IL-1, participates in the onset and development of most autoimmune diseases, such as RA and IBD (88, 188). Secondly, there is an emerging appreciation that NLRP3 inflammasome participates in adaptive immunity. Logically, NLRP3 inflammasome is involved in autoimmune diseases with adaptive immune dysfunction. For instance, IL-1 is a co-stimulatory factor and lymphocyte activating factor, which can provide signals of pro-survival and proliferation for T cells. It promotes autoreactive T cells to cause β-cell death (101). IL-1β induces migration of T cells into pancreatic islets by regulating chemotaxis (189). In RA, NLRP3 inflammasome of CD4^+^ T cells promote Th1 differentiation, which is induced by IL-1β in a caspase-1-dependent manner (83). In other autoimmune diseases, NLRP3 inflammasome can also induce differentiation and polarization of Th2, Th17, and dendritic cells (84). Thirdly, pyroptosis mediated by activated caspase-1 promotes development of autoimmune diseases. In IBD, NLRP3 inflammasome triggers pyroptosis in a caspace-1-dependent manner to cause death of histocytes such as macrophages and dendritic cells (190). The release of cellular debris reacts with immune cells, resulting in an enhanced circle of inflammation (190). Generally, IL-1β, IL-18, and pyroptosis play a function by inflammation, activating adaptive immunity and immunological regulation. Interestingly, the pathogenesis of IBD and psoriasis is mainly inflammation, and the pathogenesis of T1DM, SLE, and AITDs mainly involves adaptive immune response. Meanwhile, the pathogenesis of RA and SSC involves both above factors. So distinguishing emphasis of different autoimmune disease pathogenesis, which might involve a spectrum from auto-inflammatory to adaptive immune response, may contribute to understand the specific and accurate role of NLRP3 inflammasome.

On the other hand, besides effect of NLRP3 inflammasome pathway, NLRP3 inflammasome can promote autoimmune diseases in some other manners. In SSC, NLRP3 inflammasome activation upregulates miR-155, which participates in collagen synthesis of keratinocytes (156, 157). In summary, NLRP3 inflammasome participates in the initiation of autoimmune diseases, which serves as a checkpoint in innate immunity and adaptive immune dysfunction (**Figure 1**). Compared with clinical application focusing on downstream adaptive immune response, chemical inhibitor targeting on NLRP3 inflammasome pathway might be a more potential therapeutic strategy.

NLRP3 inflammasome is significantly increased in autoimmune diseases such as T1D, IBD, SLE, RA, SSC, psoriasis, and AITDs. However, the protective role and decrease of NLRP3 inflammasome have also been detected in T1D, IBD, SLE, and psoriasis. The controversial results may be explained by hypothesis as follows: (a) In different stages of the disease, NLRP3 inflammasome may conduct completely inverse function. For example, an experiment conducted with IRAK-M^(−/−)^ NOD mice, which is characterized by early onset and rapid progression of T1D, shows that NLRP3 inflammasome is a protective factor in the initial stage of T1D (168). (b) Despite the effect of NLRP3 inflammasome signaling pathway, NLRP3 inflammasome may also affect other bioactive substances and signaling pathways, by which it plays an opposite role. In SLE, NLRP3 inflammasome modulates TGF-β and IFN-I to conduct immunosuppressive effect (182, 191). (c) In some special pathways, NLRP3 inflammasome plays a protective role. Gastrointestinal tract is characterized by communication with external environment indirectly and existence of intestinal microflora. NLRP3 inflammasome strengthens anti-inflammatory effect to maintain intestine homeostasis but promotes modulation of T cell in a physiological condition. Therefore, deficiency and overt activation of NLRP3 inflammasome can both promote the development of IBD. In summary, future researches to identify specific mechanism of NLRP3 inflammasome in different histiocytes, disease stages and conditions are warranted.

There are several common pathways in different autoimmune diseases, such as autophagy, mitochondrial DNA, TLR4, Nrf2 pathway, Bay11-7082, P2X7 receptor, microRNA, and so on (**Table 2**). Moreover, there are specific and subtle differences between these similarly pathogenic mechanisms. For instance, the main function of miR-155 is to promote inflammation in keratinocyte of psoriasis and in macrophage of IBD; however, it drives fibrosis in fibroblasts of SSC. All these contrasts and connect in various mechanism indicate the complexity of NLRP3 inflammasome in the pathogenesis of autoimmune diseases. So, further studies are needed to focus on the comparison of mechanisms in different active sites, such as intestine and pancreatic β cell. In addition, previous studies have shown that NLRP3 inflammasome plays a protective role in physiological condition, and both the upregulation and downregulation of NLRP3 inflammasome pathway stimulated by specific factor will initiate pathogenic progress. As the mechanism of NLRP3 inflammasome assembly and downstream cytokines effect are more studied, the initiating mechanism of regulation dysfunction is of great importance in future researches. Just as avoidance of allergen to hypersensitivity, therapy focusing on initiating mechanism of regulation dysfunction will be a simple but effective therapeutic application. The inhibition of NLRP3 inflammasome pathway, such as Ginsenoside Rg1, low methoxyl pectin, and Bay11-7082, may be a potential therapeutic strategy for autoimmune diseases (**Table 2**). The inhibition involves three levels: structural proteins of NLRP3 inflammasome, cytokines, and the signaling pathway. However, the effect of some inhibitions is limited and scant. Some inhibitions cause serious side effects, and some inhibitions even show paradoxical pro-inflammatory effect. Newly plaque psoriasis could be detected in RA patients with anti-IL-1 treatment (74). It indicates that the role of NLRP3 inflammasome inhibition is complicated and associates with multiple mechanisms. Consequently, further researches are needed to focus on the following points: inhibitions that target on junction point of pathway may show precise efficacy; in addition, focusing on upstream mechanism and finding inhibition that blocks the switch of the whole pathogenic progress may bring breakthrough to therapy of autoimmune diseases.

## Conclusion

In conclusion, as a platform sensing dangerous stimuli from endogenous or exogenous environment, NLRP3 inflammasome participates in both innate and adaptive immunity *via* modulating secretion of cytokines and pyroptosis. Increasing experiments show that NLRP3 inflammasome may play different pathogenic roles in autoimmune diseases, which provides a promising therapeutic option for autoimmune diseases. Therefore, delineating a comprehensive molecular mechanism of the complex role of NLRP3 inflammasome in autoimmune diseases deserves further study.

## Author Contributions

YWZ, the main author, contributed in literature review and summary, manuscript drafting. WY assisted in reviewing literature. WL revised the manuscript. YJZ, the corresponding author, contributed in designing, reviewing, and editing the manuscript. All authors contributed to the article and approved the submitted version.

## Funding

This work was supported by National Natural Science Foundation of China (Grant No. 81700728), Science and Technology Projects of Guangzhou (Grant No. 201707010365), 2021 Medical Science Foundation of Guangdong Provincial Health Commission (Grant No. 202011179196403), 2021 Medical and Health Technology Projects of Guangzhou (Grant No. 20211A011083).

## Conflict of Interest

The authors declare that the research was conducted in the absence of any commercial or financial relationships that could be construed as a potential conflict of interest.

## Publisher’s Note

All claims expressed in this article are solely those of the authors and do not necessarily represent those of their affiliated organizations, or those of the publisher, the editors and the reviewers. Any product that may be evaluated in this article, or claim that may be made by its manufacturer, is not guaranteed or endorsed by the publisher.
